# Editorial: Basic and clinical research on neurostimulation techniques in major depressive disorder

**DOI:** 10.3389/fpsyt.2023.1287617

**Published:** 2023-10-11

**Authors:** Chaomeng Liu, Na Zhao, Yuanjun Dong

**Affiliations:** ^1^Beijing Key Laboratory of Mental Disorders, National Clinical Research Center for Mental Disorders & National Center for Mental Disorders, Beijing Anding Hospital, Capital Medical University, Beijing, China; ^2^Advanced Innovation Center for Human Brain Protection, Capital Medical University, Beijing, China; ^3^Center for Cognition and Brain Disorders, The Affiliated Hospital of Hangzhou Normal University, Hangzhou, Zhejiang, China; ^4^College of Health and Life Sciences, University of Health and Rehabilitation Sciences, Qingdao, Shandong, China

**Keywords:** major depressive disorder (MDD), clinical research, neurostimulation techniques, electroconvulsive therapy (ECT), repetitive transcranial magnetic stimulation (rTMS), transcranial alternating current stimulation (tACS)

Major depressive disorder (MDD), which is distinguished by symptoms such as diminished mood, cognitive deceleration, and decreased volitional engagement, has impacted a staggering 221 million individuals globally in 2020 (equating to 3,152.9 cases per 100,000 people) (1). Despite the escalating prevalence rates and substantial burden associated with this condition, the efficacy of depression treatment remains unsatisfactory. Conventional first-line interventions, encompassing psychotherapy and pharmacotherapy, merely attain approximately a 50% remission rate (2). Neuromodulation techniques, including transcranial magnetic stimulation (TMS), Electric convulsive therapy (ECT), vagus nerve stimulation (VNS), deep brain stimulation (DBS), transcranial alternating current stimulation (tACS), as well as the promising transcranial photobiomodulation (PBM), and transcranial ultrasound stimulation (TUS), is a biomedical engineering technology that modulates signal transmission within the nervous system, thereby regulating neuronal activity and neural networks. This technology, employing invasive or non-invasive approaches, utilizes physical or chemical methods such as electricity, magnetism, light, and ultrasound to induce specific changes in brain functions (3). Consequently, neuroregulation serves as an effective therapeutic approach for nervous system diseases and a valuable tool for investigating neural circuits and analyzing brain region functions. This Research Topic centers on the clinical advancements in the application of widely utilized neural stimulation techniques, namely ECT, tACS, and repetitive TMS (rTMS), for the management of depression.

In this Research Topic, Deng et al. conducted a cross-sectional survey in South China, utilizing a small sample size of 92 patients diagnosed with MDD and their respective caregivers (*n* = 92). The objective of the study was to examine the knowledge and attitudes of patients and caregivers toward ECT. The findings revealed that caregivers reported receiving more comprehensive information regarding the therapeutic effects (50.0 vs. 44.6%), side effects (67.4 vs. 41.3%), and risks (55.4 vs. 20.7%) of ECT compared to patients. Furthermore, a significant proportion of patients (62.0%) encountered adverse effects, with memory impairment being the prevailing complaint. Ultimately, the authors recommend that healthcare professionals establish a comprehensive health education initiative prior to administering ECT, ensuring that patients and their caregivers possess a precise comprehension of the treatment procedure, its therapeutic benefits, and potential adverse reactions.

In another study, Wang et al. conducted a study to investigate the impact of ECT on depressed patients exhibiting suicidality, utilizing resting-state functional magnetic resonance imaging (rs-fMRI). A total of 26 depressed patients with suicidality were subjected to rs-fMRI both prior to and following 8–12 sessions of ECT. The assessment of whole brain function involved the measurement of amplitude of low-frequency fluctuations (ALFF), fractional amplitude of low-frequency fluctuations (fALFF), and regional homogeneity (ReHo). At the outset, it was observed that patients displayed diminished ALFF in the right postcentral and precentral gyrus, along with reduced fALFF in the right supramarginal and postcentral gyrus, left superior frontal gyrus (SFG), as well as the superior and middle temporal gyrus, in comparison to the control group consisting of healthy individuals. Subsequent to ECT, a noteworthy augmentation in fALFF was observed in the left SFG and orbital frontal cortex (OFC), which exhibited a significant inverse correlation with the decline in depression scores. According to their findings, it is believed that the left SFG and OFC could potentially have a significant impact on the mechanism of ECT for addressing suicidality. The observed reduction in fALFF in the left SFG and OFC may serve as a plausible mechanism by which ECT effectively alleviates suicidality in individuals suffering from depression.

A randomized controlled trial (RCT) conducted by Pan et al. aimed to examine the impact of rTMS on cognitive function among elderly individuals diagnosed with late-onset depression (LOD). The study involved the administration of either active or sham rTMS targeting the left dorsolateral prefrontal cortex, with a frequency of 10 Hz rTMS and an intensity set at 120% of the motor threshold. Cognitive function assessments were conducted at baseline, the conclusion of the 4-week treatment period, and during the 4-week follow-up period. The findings of this study indicate that rTMS may have a positive impact on cognitive functioning, specifically immediate memory and attention, in patients with LOD. These results suggest that incorporating rTMS into the treatment options for LOD in clinical practice could be beneficial. However, it is important to exercise caution when interpreting these findings due to the relatively small sample size, which limits the ability to conduct subgroup analysis or generalize the results to a larger population.

Finally, Zheng et al. conducted a meta-analysis of RCTs to comprehensively examine the therapeutic efficacy and tolerability of tACS in the treatment of individuals diagnosed with MDD. The study included a total of four RCTs, encompassing five active treatment arms, which involved 224 participants diagnosed with MDD. The participants were divided into two groups: those receiving active tACS (*n* = 117) and those receiving sham tACS (*n* = 107). The meta-analysis of depressive symptoms following tACS administration revealed a significant advantage of active tACS over sham tACS. Further research is necessary to confirm the efficacy and safety of tACS in ameliorating depressive symptoms among depressed patients.

In conclusion, the articles encompassed within this topic contribute supplementary evidence derived from clinical research endeavors to the existing body of literature concerning neurostimulation techniques in MDD. Nevertheless, forthcoming investigations ought to prioritize the resolution of the subsequent concerns. Firstly, the refinement of therapeutic targets and stimulation parameters necessitates further attention, a task that could be enhanced through the utilization of neuroimaging and electroencephalography (EEG) methodologies. Secondly, the utilization of neurostimulation techniques is hindered by various factors, including the adverse effects and limited convenience associated with these methods. For instance, ECT may result in temporary memory impairment, TMS exhibits a short-lived therapeutic effect, and both DBS and VNS necessitate the implantation of stimulators. Lastly, the underlying treatment mechanism remains unclear; however, employing animal models of mental disorders, molecular biology, neuroimaging, and electrophysiological techniques may contribute to a more comprehensive comprehension of the principles governing neurostimulation techniques.

## Author contributions

CL: Writing—original draft. NZ: Writing—review and editing. YD: Supervision, Writing—review and editing.
